# Role of the phloem in the biochemistry and ecophysiology of benzylisoquinoline alkaloid metabolism

**DOI:** 10.3389/fpls.2013.00182

**Published:** 2013-06-11

**Authors:** Eun-Jeong Lee, Jillian M. Hagel, Peter J. Facchini

**Affiliations:** Department of Biological Sciences, University of CalgaryCalgary, AB, Canada

**Keywords:** benzylisoquinoline alkaloid, phloem, laticifer, sieve element, plant defense

## Abstract

Benzylisoquinoline alkaloids (BIAs) are a diverse group of biologically active specialized metabolites produced mainly in four plant families. BIA metabolism is likely of monophyletic origin and involves multiple enzymes yielding structurally diverse compounds. Several BIAs possess defensive properties against pathogenic microorganisms and herbivores. Opium poppy (*Papaver somniferum*: Papaveraceae) has emerged as a model system to investigate the cellular localization of BIA biosynthesis. Although alkaloids accumulate in the laticifer cytoplasm (latex) of opium poppy, corresponding biosynthetic enzymes and gene transcripts are localized to proximal sieve elements and companion cells, respectively. In contrast, BIA metabolism in the non-laticiferous meadow rue (*Thalictrum flavum*; Ranunculaceae) occurs independent of the phloem. Evidence points toward the adoption of diverse strategies for the biosynthesis and accumulation of alkaloids as defensive compounds. Recruitment of cell types involved in BIA metabolism, both within and external to the phloem, was likely driven by selection pressures unique to different taxa. The biochemistry, cell biology, ecophysiology, and evolution of BIA metabolism are considered in this context.

## Introduction

Alkaloids are low-molecular weight, nitrogenous specialized metabolites occurring in approximately 20% of plant species. Many of the ~12,000 structurally elucidated alkaloids show potent biological activity. In particular, the pharmacological properties of benzylisoquinoline alkaloids (BIAs) have been exploited for millennia, and modern medicine continues to rely on plant-derived compounds such as the analgesics morphine and codeine. BIAs such as berberine and sanguinarine possess antimicrobial activity, whereas others such as noscapine are potentially antineoplastic (Barken et al., 2008). Papaverine and (+)-tubocurarine are effective vasodilators and muscle relaxants, respectively, and the morphine precursor thebaine is used for the synthesis of semi-synthetic drugs such as oxycodone, naltrexone, and buprenorphine. Ironically, more is understood about the effects of alkaloids on humans than the roles of these compounds in the plants that produce them. Although not considered essential for normal growth and development, BIAs likely play key roles in the defense of plants against herbivores and pathogens. Compared with our rather superficial appreciation for the ecophysiology of compounds such as morphine and noscapine, the biochemistry of BIA biosynthesis is well-established (Hagel and Facchini, 2013). An impressive array of biosynthetic enzymes function to (1) rearrange the core 1-benzylisoquinoline backbone and (2) add or modify functional moieties, yielding a vast diversity of approximately 2500 known BIAs originating from a common intermediate (Ziegler and Facchini, 2008). Phylogenetic data supports a common origin for these alkaloids in certain families of the order Ranunculales (Liscombe et al., 2005). However, remarkable differences are apparent with respect to BIA biosynthesis and storage. In opium poppy (*Papaver somniferum*: Papaveraceae), sieve elements and specialized laticifers of the phloem produce and accumulate BIAs, respectively (Bird et al., 2003; Samanani et al., 2006). In contrast, phloem tissues are not involved in the biosynthesis or accumulation of BIAs in meadow rue (*Thalictrum flavum*: Ranunculaceae) (Samanani et al., 2005). The inclusion or exclusion of phloem or other tissues in BIA metabolism was likely driven by several factors including the potentially independent emergence of laticifers and the inherent biological activities of a persistently changing arsenal of BIAs.

## Defensive properties of BIAs

Numerous studies have supported defensive roles for BIAs in plants, some of which possess anti-herbivory, antifungal and/or antibacterial properties. Several structural subgroups have been implicated, including protoberberine, benzophenanthridine, protopine, aporphine, bisbenzylisoquinoline, pthalideisoquinoline, and morphinan alkaloids (Table 1). For example, berberine exhibits potent anti-herbivory activity toward a variety of insects including gypsy moth larvae (*Lymantria dispar*) (Shields et al., 2008), fourth instar larvae of fall webworms (*Hyphantria cunea*), adult Alder leaf beetles (*Agelastica coerulea*) (Park et al., 2000) and fruit flies (*Drosophila melongaster*) (Sellier et al., 2011). Coupled with reduced larval growth and survival rates among generalist pests (Krug and Proksch, 1993), such activity has prompted consideration of berberine as a commercial insecticide (Shields et al., 2008). Quaternary ammonium salts of certain protoberberine and benzophenanthridine (e.g., sanguinarine and chelerythrine) alkaloids also show antifungal and antibacterial activities toward economically important plant pathogens (Liu et al., 2009). Antimicrobial properties are also associated with aporphine alkaloids (Villar et al., 1987; Zhang et al., 2012), whereas the protopine alkaloid hunnemanine inhibits spore germination of phytopathogenic fungi (Singh et al., 2009). The defensive properties of BIAs not only target microorganisms and insects, but also larger pests including mammals, which seem prone to the bitterness of alkaloids. For example, the buffy-headed marmoset (*Callithrix flaviceps*), a primate native to South America, avoids the BIA-rich fleshy fruit of the rainforest fevertree (*Siparuna guianensis*) when consuming the nutritious seeds (Simas et al., 2001). Roots of California poppy (*Eschscholzia californica*) with high alkaloid content are less palatable to gophers (Geomyidae) compared with low-alkaloid cultivars (Watts et al., 2011). In opium poppy subjected to mechanical damage the rapid formation and incorporation of bismorphine (Table 1) into the cell wall was suggested to serve a defensive function by decreasing susceptibility to hydrolysis by pectinases (Morimoto et al., 2001).

**Table 1:** **Selected BIAs with established or putative defensive properties**.

**Compound**		**Structural type**	**Activity**	**References**
Berberine	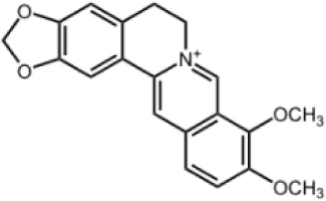	Protoberberine	Feeding deterrent	Krug and Proksch, 1993; Schmeller et al., 1997; Park et al., 2000; Tims and Batista, 2007; Shields et al., 2008; Sellier et al., 2011
			Antifungal	
			Antibacterial	
			Antiviral	
Hydrastine	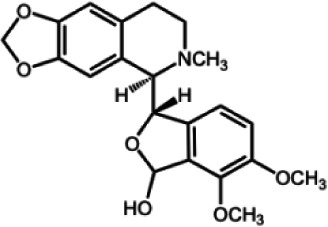	Pthalideisoquinoline	Antifungal	Goel et al., 2003; Tims and Batista, 2007
Hunnemanine	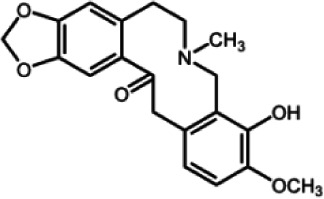	Protopine	Antifungal	Singh et al., 2009
Chelerythrine	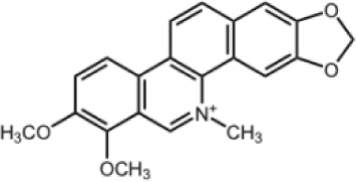	Benzophenanthridine	Antifungal	Liu et al., 2009
Papaverine	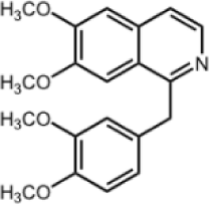	Benzylisoquinoline	Feeding deterrent	Sellier et al., 2011
Sanguinarine	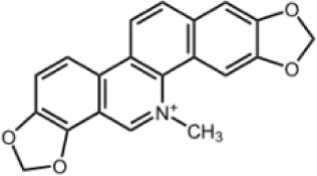	Benzophenanthridine	Antifungal	Schmeller et al., 1997; Liu et al., 2009
			Antibacterial	
			Antiviral	
Tabienine B	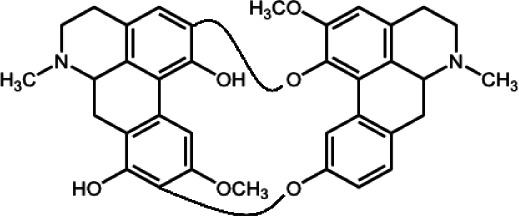	Bisbenzylisoquinoline	Insecticidal^a^	Quevedo et al., 2011
Anolobine	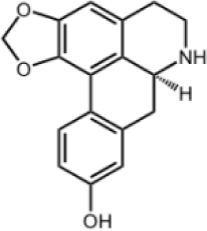	Aporphine	Antibacterial	Villar et al., 1987
			Antifungal	
Bismorphine	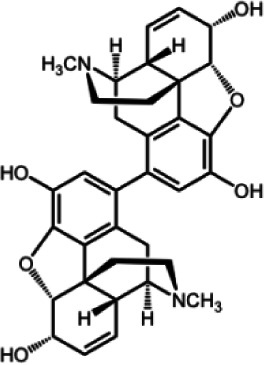	Morphinan	Cell wall strengthening^b^	Morimoto et al., 2001
Liriodenine	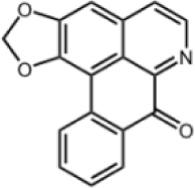	Aporphine	Antibacterial	Villar et al., 1987; Costa et al., 2010
			Antifungal	

a Larvicidal.

b Reduces cell wall hydrolysis by pectinases.

## Role of phloem in the production and storage of BIAs

The biochemistry of BIA metabolism has recently been reviewed in detail (Hagel and Facchini, 2013). Briefly, BIA biosynthesis begins with the condensation of two tyrosine derivatives, dopamine and 4-hydroxyphenylacetaldehyde to yield the central intermediate (*S*)-norcoclaurine. The key branch-point intermediate (*S*)-reticuline is formed from (*S*)-norcoclaurine via 6-*O*-methylation, *N*-methylation, aromatic ring hydroxylation and 4'-*O*-methylation. Protoberberine, protopine, benzophenanthridine and pthlalideisoquinoline alkaloids are derived from (*S)-reticuline* via flavoprotein oxidase-catalyzed oxidative cyclization. Pathway enzymes leading to berberine and sanguinarine (Table 1) have been fully isolated. Berberine biosynthesis involves *O*-methylation followed by two successive oxidation steps yielding a methylenedioxy bridge and a quaternary ammonium salt. Sanguinarine production requires the incorporation of two methylenedioxy bridges, *N*-methylation, and three subsequent oxidations mediating molecular rearrangement and quaternary alkaloid formation. Phthalideisoquinoline alkaloid metabolism is not well-understood, although recent efforts have provided a metabolic framework and candidate biosynthetic genes involved in noscapine biosynthesis (Dang and Facchini, 2012; Winzer et al., 2012). An enzyme forming an aporphine bridge in (*S*)-reticuline was isolated from Japanese goldthread (*Coptis japonica*) (Ikezawa et al., 2008) although downstream enzymes have not been characterized. One *O*-methyltransferase involved in the biosynthesis of papaverine (Table 1) is not known (Desgagné-Penix and Facchini, 2012). In morphine biosynthesis, (*S*)-reticuline is epimerized to (*R*)-reticuline, which undergoes C-C phenol coupling to the promorphinan alkaloid salutaridine. Subsequent carbonyl reduction and *O*-acetylation yield thebaine, which is converted to morphine by two oxidative *O-demethylations* and keto reduction.

### Phloem cells work together in opium poppy

The order Ranunculales is the first diverging clade of the eudicots containing seven families representing approximately 3350 species, most of which are associated with the families Ranunculaceae, Papaveraceae, Berberidaceae, and Menispermaceae (Forest and Chase, 2009). The Papaveraceae form the earliest diverging group within the Ranunculales and includes the genus *Papaver*, all species of which produce latex containing BIAs. Opium poppy accumulates many different structural subgroups of BIAs and has emerged as a model system to investigate alkaloid metabolism (Facchini and De Luca, 2008). BIAs accumulate in laticifers found adjacent or proximal to sieve elements of the phloem in all organs of opium poppy. Morphinan, pthalideisoquinoline (i.e., noscapine) and 1-benzylisoquinoline (i.e., papaverine) alkaloids are major compounds in the latex of aerial organs, whereas the benzophenanthridine sanguinarine is predominant in roots. Latex is the cytoplasm of laticifers, which are specialized cells with either articulated or non-articulated morphologies (Metcalfe, 1967; Hagel et al., 2008). Articulated laticifers consist of longitudinal cellular chains with perforated or entirely degraded adjacent walls. Non-articulated laticifers arise from a single cell that continuously by elongation and ramification through plant tissues (Nessler and Mahlberg, 1977). Both laticifer types are generally associated with vascular tissues and contain a full complement of cellular organelles that often includes large vesicles into which alkaloids are sequestered (Hagel et al., 2008). Opium poppy laticifers are articulated, undergo extensive anastomosis (adjacent cell wall degradation), contain many vesicles, and are confined almost entirely to the phloem (Nessler and Mahlberg, 1979). These specialized alkaloid warehouses are formed in the procambium and later in the vascular cambium, thereby sharing a common origin with sieve elements and companion cells.

**Figure 1:**
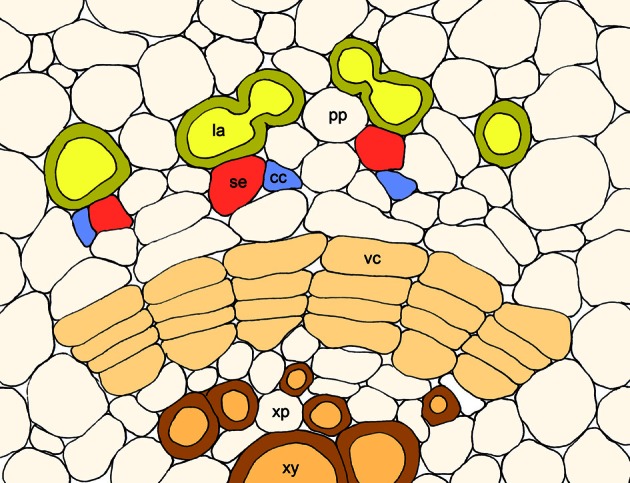
**Schematic representation of the multicell model for the biosynthesis and accumulation of BIAs in opium poppy involving the coordinated participation of companion cells (cc), sieve elements (se), and laticifers (la)**. Biosynthetic enzymes are synthesized in companion cells and transported to sieve elements, where alkaloid biosynthesis occurs. The bulk of synthesized alkaloids are stored in neighboring laticifer networks. Other abbreviations: phloem parenchyma (pp), vascular cambium (vc), xylem parenchyma (xp), and xylem vessels (xy).

Although laticifers were initially thought to function in both the synthesis and storage of BIAs, more recent localization studies have demonstrated the involvement of neighboring phloem tissues (Bird et al., 2003; Weid et al., 2004; Samanani et al., 2006; Lee and Facchini, 2010). The current multi-cell model for BIA biosynthesis and accumulation in opium poppy is illustrated in Figure 1. All known enzymes involved in the formation and conversion of (*S*)-norcoclaurine to (*S*)-reticuline, and catalyzing one early and one intermediate step in the sanguinarine and morphine branch pathways, respectively, have been localized to sieve elements by immunofluorescence labeling. As expected, corresponding gene transcripts were localized to companion cells by *in situ* RNA hybridization. The localization of BIA biosynthesis to sieve elements implicates the intercellular transport of pathway intermediates or products. Whether BIA trafficking is symplastic involving plasmodesmata, or apoplastic implicating transporters or channels (Figure 1) is not known. The characterization of an ATP-binding cassette (ABC) transporter in Japanese goldthread capable of berberine transport provides some insight into a possible mechanism (Shitan et al., 2003).

### Phloem tissues are not required in some plants

Despite the close phylogenetic relationship between corresponding biosynthetic enzymes in meadow rue and opium poppy (Liscombe et al., 2005; Samanani et al., 2005) different cell types are involved in BIA biosynthesis. Notably, meadow rue does not contain laticifers and accumulates alkaloids such as berberine (Table 1) in roots and rhizomes. Protoberberine alkaloids are restricted to endodermal cells upon initiation of secondary growth in roots and are distributed throughout the pith and cortex in rhizomes (Samanani et al., 2005). Opium poppy and meadow rue show the paradigmatic recruitment of multiple cell types for alkaloid biosynthesis and accumulation. In meadow rue roots, BIA biosynthetic gene transcripts were localized to the pericycle (the innermost layer of the cortex) and adjacent cortical cells, whereas gene transcript accumulation was restricted to the protoderm of rhizome leaf primordia. The cell-type specific localization of BIA biosynthesis and accumulation are, thus, temporally and spatially separated in meadow rue. Unlike opium poppy, BIA biosynthesis and accumulation occurs outside of the vascular system, although localization of key enzymes to the endodermis and pericycle likely provides convenient access to the precursor tyrosine from the phloem.

Opium poppy and meadow rue currently provide the most accurate models highlighting variations in the cellular localization of BIA metabolism. However, other studies suggest the occurrence of additional compartmentalization strategies in terms of BIA biosynthesis and storage. For example, tissue-specific profiling of BIAs in the stems of *Sinomenium acutum* (Menispermaceae) using laser micro-dissection coupled with liquid chromatography-mass spectrometry showed a wide distribution of alkaloids, especially within the outer cortical regions, phloem and xylem (Yi et al., 2012). BIAs were also reported to accumulate outside of phloem tissues in the fruit of plants, such as plume poppies (*Macleaya* spp.; Papaveraceae) (Zeng et al., 2013) and fevertree (*Siparuna guianensis*: Siparunaceae) (Simas et al., 2001).

## Evolution and ecological roles of phloem in BIA metabolism

The predominance of BIAs in basal angiosperms suggests an ancient evolutionary origin for this group of specialized metabolites (Facchini et al., 2004). A monophyletic origin is supported by the extensive sequence homology among enzymes operating at conserved points in BIA metabolic networks found in plants belonging to different families. However, despite widespread similarities in the biosynthetic machinery, involvement of the phloem in opium poppy compared with the disengagement of vascular tissues in meadow rue suggests the migration of previously established BIA biosynthetic pathways between cell types. It is reasonable to suggest that widely distributed alkaloids, such as berberine and sanguinarine, were selected based on inherent anti-herbivory and antimicrobial properties. It is also conceivable that adaptive evolution was the driving force behind the differential cellular location of BIA metabolism in various plants. Access of herbivores and pathogens to specific cell types, tissues and organs such as sieve elements, laticifers, epidermis, and fruits likely had a profound effect on the sites of defensive alkaloid accumulation and, thus, the localization of BIA biosynthesis.

The localization of defensive metabolites to phloem is not restricted to BIAs. Whereas the primary function of phloem is the long-distance translocation of nutrients and information molecules, certain phloem cell types contribute to the synthesis, distribution and release of numerous defense compounds. In some plants, sieve element sap contains proteins and/or metabolites that deter herbivores and are toxic to pathogens (Hagel et al., 2012). Examples of defensive compounds found in phloem sap include quinolizidine alkaloids (Lee et al., 2007), ketone steroids (Behmer and Nes, 2003; Janson et al., 2009; Behmer et al., 2010), sulfur-containing metabolites such as glucosinolates (Halkier and Gershenzon, 2006; Hopkins et al., 2009), and perhaps cyanogenic glycosides (Jørgensen et al., 2005, 2011). Phloem transport of defensive compounds such as glucosinolates is often coupled with their biosynthesis, which occurs in specialized cells proximal to sieve elements. Arguably, the localization of defensive metabolites to sieve elements provides a deterrent to sucking insects attempting to access the sugar-rich phloem sap. Certain pests such as aphids have developed counter-strategies to overcome phloem defense mechanisms (Hagel et al., 2012). Although BIA biosynthesis occurs in the sieve elements of opium poppy, efforts to detect BIAs in phloem sap have not yet been pursued. It is possible a broad spectrum of alkaloids, or a specific subgroup thereof, is maintained in sieve elements as a deterrent to phloem-feeding insects. Nevertheless, the copious quantity of alkaloids sequestered to latex suggests that transport from sieve elements to laticifers confers an important selective advantage in opium poppy.

### Emergence and role of laticifers

Laticifers are generally considered a recently evolved cell type, and likely arose independently in different plant taxa (Hagel et al., 2008). However, the ubiquitous distribution of laticifers in families such as the Papaveraceae suggests isolated pockets of monophylogeny. Latex often contains specialized metabolites, including alkaloids, terpenoids, cardiac glycosides, lignans, cannabinoids, and tannins (Konno, 2011; Mithöfer and Boland, 2012). Beyond the defensive properties of such metabolites, the glue-like consistency of the latex itself appears to have a defensive function by coating the mouthparts of foraging herbivores (Hagel et al., 2008). The emergence of latex has undoubtedly contributed to the arsenal of defenses in many plants, although laticifers are not always involved in the storage of potentially defensive metabolites. For example, the pentacyclic quinoline alkaloid camptothecin accumulates in the parenchyma and/or epidermal cells of the roots, stems and leaves of Chinese happy tree (*Camptotheca acuminata*) (Pasqua et al., 2004), but is absent from laticifers (Monacelli et al., 2005). In hemp (*Cannabis sativa*), cannabinoids accumulate mostly in glandular trichomes rather than in the non-articulated laticifers (Page and Nagel, 2006). In the plume poppy (*Macleaya cordata*), protopine alkaloids are predominant in roots and leaves, whereas sanguinarine accumulation is highest in fruits (Table 1) (Zeng et al., 2013). Interestingly, laticifers are notably absent in the BIA-rich fruit tissues of *M. cordata*. Comparison of different plume poppy species and accessions revealed remarkable flexibility in alkaloid storage sites, although the cellular localization of BIA biosynthesis is unknown.

The emergence of laticifers in plume poppies was potentially not exploited for the purpose of BIA accumulation to the same extent as the profound role of latex as an alkaloid storage site in opium poppy. Basic BIA biosynthesis was likely established prior to the evolution of laticifers as unique phloem cell type in the Papaveraceae. Progenitors of the Papaveraceae might have harbored a capacity for BIA biosynthesis in sieve elements or other phloem cell types, and the emergence of laticifers provided a convenient site for the accumulation of copious alkaloid quantities, which potentially enhanced evolutionary fitness. Latex generally exhibits positive turgor and exudes upon tissue damage such as that incurred during herbivory (Hagel et al., 2008). Alternatively, laticifers in ancestors of the Papaveracae would represent only one option for the establishment of a high-capacity alkaloid accumulation site since other cell or tissue types exposed to pathogens and herbivores and capable of alkaloid storage could be available. Fleshy fruit is a common target of herbivores and would represent an effective storage site for defensive BIAs; thus, it is not surprising that some species might have adopted this strategy (Zeng et al., 2013). Clearly, the evolutionary and ecological relationship between plant-herbivore interactions and BIA metabolism requires further investigation.

### Phloem involvement: to be or not to be

The emergence of laticifers within the Papaveraceae arguably provided a unique opportunity for the sequestration of preexisting alkaloids. However, alternative strategies for the compartmentalization of BIA biosynthesis and accumulation were required for plants in which laticifers did not evolve. In case of meadow rue, phloem tissues appear entirely excluded from BIA metabolic processes with the likely exception of supplying the precursor tyrosine (Samanani et al., 2005). However, the current lack of phloem involvement in meadow rue BIA metabolism does not preclude the possibility that the pathway migrated from phloem to non-phloem tissues over the course of evolution. If the capacity for BIA biosynthesis originated in sieve elements, relocation of the pathway to non-phloem tissues might have occurred in support of a specific ecophysiological advantage. The accumulation of berberine in the endodermis and pericycle of meadow rue roots creates a defensive boundary (Samanani et al., 2002). An effective solution to the challenge of invasion by soil-born pathogens might have involved an outward migration of BIA metabolism from phloem to surrounding tissues in members of the Ranunculaceae. Interestingly, endodermis and pericycle have also been implicated in the biosynthesis and accumulation of other defensive metabolites, such as tropane alkaloids (Hashimoto et al., 1991; Nakajima and Hashimoto, 1999; Suzuki et al., 1999).

Alternatively, BIA biosynthesis might have originated in non-phloem tissues with migration of pre-existing pathways to sieve elements in plants such as opium poppy. However, the origin of BIA metabolism within vascular tissues can be argued on the basis that the establishment of biosynthetic capacity proximal to a variety of cell types provides a more versatile platform for pathway migration to tissues capable of deploying the advantage conferred by the capacity to produce defensive compounds. For example, sieve elements would provide access to roots, fruits, latex and other plant organs and tissues. Migration of alkaloid biosynthesis to proximal tissues could hypothetically occur under species-specific selection pressures resulting in a variety of different localization patterns in different taxa.

## Conclusions and perspectives

The questions of how, why and where BIA biosynthesis originated remains a matter of speculation. Evidence supporting the monophyletic origin of BIA metabolism in plants strongly suggests migration of the biosynthetic machinery between different cell types during the course of evolution in the Ranunculales. The defensive properties of certain BIAs were likely driving forces for such migration, resulting in the placement of specific alkaloids in cellular locations that would confer optimal selective advantages. In opium poppy and likely other members of the Papaveraceae, phloem is central to BIA biosynthesis and accumulation. In contrast, phloem tissues do not appear to play a role in BIA metabolism in meadow rue and likely other members of the Ranunculaceae. The relatively few species studied so far highlight the diversity of localization strategies. Continued investigation of the cell-specific localization of BIA metabolism in and taxonomically expanded group of plants will further provide further insights into the remarkable evolution and ecology of plant specialized metabolism.

### Conflict of interest statement

The authors declare that the research was conducted in the absence of any commercial or financial relationships that could be construed as a potential conflict of interest.
